# Variation in MicroRNA Expression Profile of Uterine Leiomyoma with Endometrial Cavity Distortion and Endometrial Cavity Non-Distortion

**DOI:** 10.3390/ijms19092524

**Published:** 2018-08-25

**Authors:** Yong Jin Kim, Yoon Young Kim, Jung Ho Shin, Hoon Kim, Seung-Yup Ku, Chang Suk Suh

**Affiliations:** 1 Department of Obstetrics and Gynecology, Korea University Guro Hospital, Korea University College of Medicine, Seoul 110-744, Korea; zinigo@gmail.com (Y.J.K.); shinjh@korea.ac.kr (J.H.S.); 2 Department of Obstetrics and Gynecology, Seoul National University Hospital, Seoul National University College of Medicine, 28 Yonkeun-dong, Chongno-gu, Seoul 110-744, Korea; yoonykim96@gmail.com (Y.Y.K.); obgyhoon@gmail.com (H.K.); suhcs@snu.ac.kr (C.S.S.)

**Keywords:** uterine leiomyoma, microRNA, endometrial cavity distortion

## Abstract

The expression profile of microRNA (miRNA) in uterine leiomyoma (UL) cells is different from that in normal uterine myometrial (UM) cells. The effect of UL cells on uterine receptivity might vary according to their ability to distort the uterine endometrial cavity. However, the variation in miRNA expression profiles between endometrial cavity-distorting leiomyoma (ECDL) and endometrial cavity non-distorting leiomyoma (ECNDL) cells remains unknown. This study aimed to elucidate whether the expression profile of miRNAs in ECDL cells is dissimilar to that of ECNDL cells in uterus. Pelviscopic myomectomy was performed to obtain tissue samples of UL and their corresponding normal UM tissues (matched) from patients with UL (*n* = 26), among whom women with ECNDL and ECDL numbered 15 and 11, respectively. The relative expression of hsa-miR-15b, -29a, -29b, -29c, -197, and -200c as well as the candidate target genes in UL cells was compared to those in the matched UM cells using qRT-PCR to assess their ability to cause ECD. The spatial expression of miRNAs and target genes in the UL tissues was analyzed using in situ hybridization. Target gene expression was analyzed using qPCR after transfection with the mimics and inhibitors of miRNAs in UL cells. The relative expression level of miR-15b was upregulated, and the relative expression levels of miR-29a, -29b, -29c, -197, and -200c were downregulated in UL cells compared to those in UM cells. The relative expression levels of progesterone receptor, estrogen receptor, and matrix metalloproteinases (MMPs) were upregulated in UL cells compared to those in UM cells. The relative expression levels of miR-29c and -200c were downregulated, and the relative expression levels of estrogen receptor, MMPs and tissue inhibitors of metalloproteinases (TIMPs) were upregulated in ECDL cells compared to those in ECNDL cells. The expression profile of miRNAs in UL cells varied with respect to the occurrence or absence of endometrial cavity distortion. The biochemical properties of UL might be regulated by miRNAs in order to alter their effect on structural homeostasis of the uterus.

## 1. Introduction

Uterine leiomyomas (ULs) are the most common benign tumors of the female reproductive tract in humans [1]. Although most ULs do not exhibit symptoms, symptomatic tumors cause chronic pelvic pain, abnormal uterine bleeding, and infertility owing to uterine factor [2]. In particular, ULs that cause anatomical disruption of the normal uterine endometrial structure might affect the implantation and development of embryos [3,4]. A few studies reported an improvement in the pregnancy outcome after surgically treating ULs with endometrial cavity distortion (ECD) using myomectomy [5,6].

MicroRNAs (miRNAs) are non-coding RNA molecules that are endogenously produced and post-transcriptionally regulate gene expression by inhibiting translation or cleaving the complementary target messenger RNAs (mRNAs) [7]. miRNAs were found to play important roles in intracellular signaling, apoptosis, metabolism, organogenesis, and embryonic development [8,9,10,11,12,13,14,15,16]. Several recent studies implicated the potential regulatory functions of a few specific miRNAs in order to elucidate the pathogenesis of UL. These studies compared the expression levels of specific miRNAs in uterine myometrium (UM) and UL tissues [17,18,19]. However, the variation in miRNA expression profile of UL tissue according to accompanying ECD remains unknown, although its clinical implication could be significant.

This study aimed to evaluate the variation between the miRNA expression profiles in UL tissues of patients with ECD and endometrial cavity non-distortion (ECND) using UM tissues as matched controls. To elucidate the regulatory function of miRNAs on the candidate target genes, analogues of miRNAs were transfected into UL cells under in vitro culture conditions.

## 2. Results

### 2.1. Characteristics of the Patients and Uterine Leiomyoma (UL) Tissues

Significant variations in patient characteristics such as age, UL size, and main symptoms were not observed between the subjects with ECDL and ECNDL (Table 1). Both types of UL tissues exhibited similar distribution with respect to grade of hardness (Table 2).

### 2.2. The Expression Profiles of miRNAs in UL Cells

**Figure 1:**
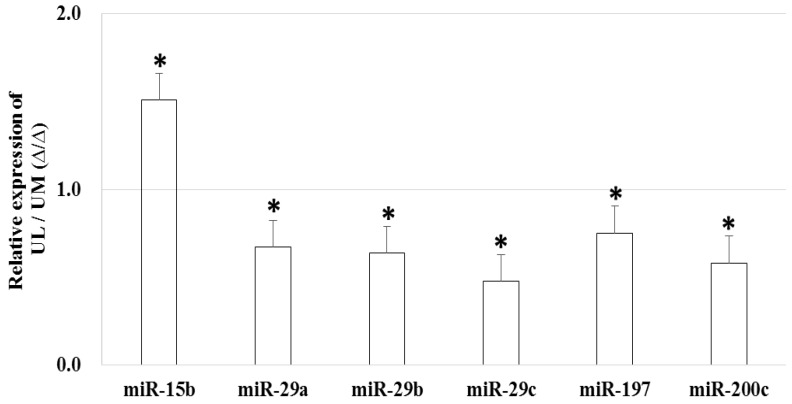
The relative miRNA expression in uterine leiomyoma cells compared to uterine myometrial cells (* *p* < 0.05).

**Figure 2:**
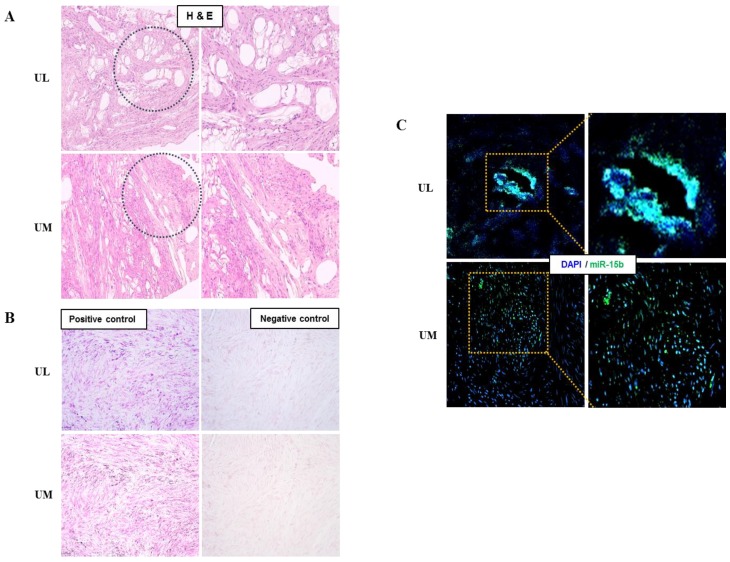
Detection of miRNA expression in human uterine leiomyoma tissue using in situ hybridization. (**A**) Observation of leiomyoma tissue (H&E staining, ×100). (**B**) Leiomyoma positive and negative control (Fast red staining, ×200). (**C**) Evaluation of miR-15b expression in leiomyoma tissue using miR-15b in situ hybridization (green, ×200).

The relative expression level of miR-15b (1.509-fold, *p* = 0.044) was upregulated and the relative expression level of miR-29a (0.671-fold, *p* = 0.008), miR-29b (0.639-fold, *p* < 0.001), miR-29c (0.479-fold, *p* < 0.001), miR-197 (0.751-fold, *p* = 0.005), and miR-200c (0.581-fold, *p* < 0.001) were downregulated in UL cells compared to in matched UM cells (Figure 1). The result of in situ hybridization to visualize miRNA expression demonstrated that miR-15b was localized in the UL tissue (Figure 2).

### 2.3. The Expression Levels of Candidate Target Genes in UL Cells

**Figure 3:**
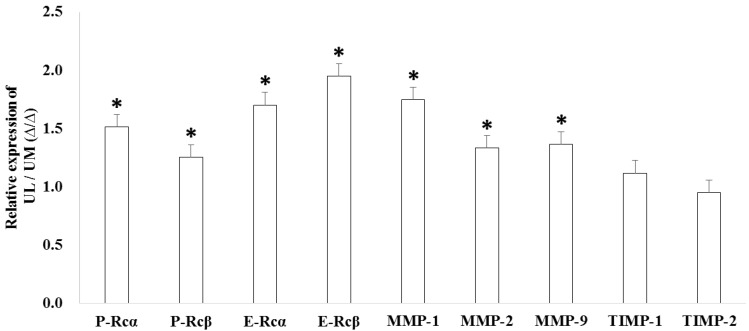
The relative candidate target gene expression in uterine leiomyoma cells compared to uterine myometrial cells (* *p* < 0.05).

**Figure 4:**
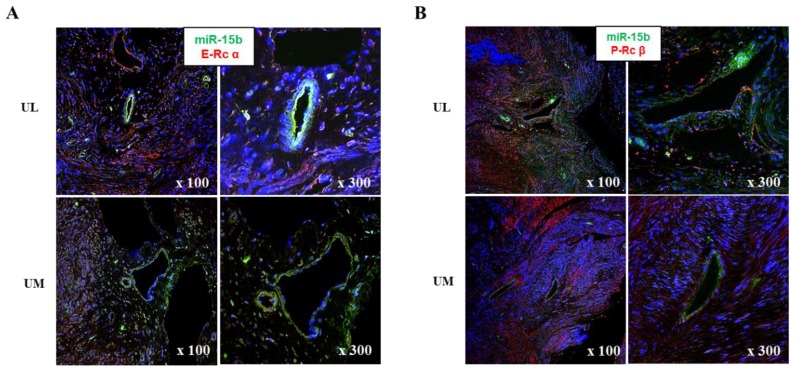
Analyses of target gene expression in myoma tissue. (**A**) Co-localization of miR-15b with estrogen receptor α. (**B**) Co-localization of miR-15b with progesterone receptor β.

The relative expression levels of progesterone receptor α (P-Rcα, 1.518-fold, *p* = 0.034), progesterone receptor β (P-Rcβ, 1.257-fold, *p* = 0.040), estrogen receptor α (E-Rcα, 1.704-fold, *p* < 0.001), estrogen receptor β (E-Rcβ, 1.951-fold, *p* < 0.001), matrix metalloproteinase-1 (MMP-1, 1.750-fold, *p* < 0.001), MMP-2 (1.336-fold, *p* = 0.025), and MMP-9 (1.367-fold, *p* = 0.037) were upregulated in UL cells compared to in matched UM cells (Figure 3). The results of immunofluorescence staining exhibited co-localization of miR-15b with E-Rcα and -Rcβ (Figure 4).

### 2.4. The Expression Levels of Candidate Target Genes after miRNA Transfection into UL Cells

**Figure 5:**
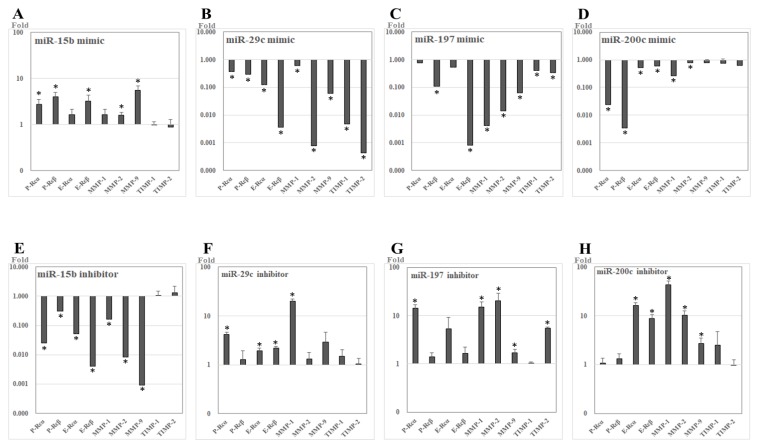
The relative expression of candidate target genes of studied miRNAs after transfection with miRNA analogues into leiomyoma cells cultured in vitro (RT-PCR). (**A**–**D**) miR-mimics. (**E**–**H**) miR-inhibitors. * *p* <0.05.

After transfection of miR-15b mimic into UL cells that were cultured in vitro, the relative expression levels of P-Rcα (2.736-fold, *p* = 0.020), P-Rcβ (4.011-fold, *p* = 0.009), E-Rcβ (3.265-fold, *p* = 0.019), MMP-2 (1.610-fold, *p* = 0.020), and MMP-9 (5.587-fold, *p* = 0.005) were upregulated compared to in control UL cells. After treatment with miR-15b inhibitor in UL cells, the relative expression levels of P-Rcα (0.025-fold, *p* = 0.002), P-Rcβ (0.304-fold, *p* = 0.031), E-Rcα (0.052-fold, *p* = 0.006), E-Rcβ (0.004-fold, *p* = 0.001), MMP-1 (0.163-fold, *p* = 0.003), MMP-2 (0.008-fold, *p* = 0.001), and MMP-9 (0.001-fold, *p* < 0.001) were downregulated compared to in control UL cells (Figure 5).

After transfection of miR-29c mimic into UL cells, the relative expression levels of P-Rcα (0.372-fold, *p* = 0.008), P-Rcβ (0.289-fold, *p* = 0.016), E-Rcα (0.122-fold, *p* = 0.008), E-Rcβ (0.004-fold, *p* = 0.001), MMP-1 (0.594-fold, *p* = 0.043), MMP-2 (0.001-fold, *p* = 0.014), MMP-9 (0.060-fold, *p* < 0.001), tissue inhibitor of metalloproteinases-1 (TIMP-1, 0.005-fold, *p* < 0.001), and TIMP-2 (<0.001-fold, *p* < 0.001) were downregulated compared to in control UL cells. After treatment with miR-29c inhibitor in UL cells, the relative expression levels of P-Rcα (4.195-fold, *p* = 0.005), E-Rcα (1.940-fold, *p* = 0.010), E-Rcβ (2.203-fold, *p* = 0.003), and MMP-1 (20.271-fold, *p* < 0.001) were upregulated compared to in control UL cells.

After transfection of miR-197 mimic into UL cells, the relative expression levels of P-Rcβ (0.109-fold, *p* < 0.001), E-Rcβ (0.001-fold, *p* < 0.001), MMP-1 (0.004-fold, *p* < 0.001), MMP-2 (0.014-fold, *p* = 0.002), MMP-9 (0.065-fold, *p* < 0.001), TIMP-1 (0.412-fold, *p* = 0.028), and TIMP-2 (0.336-fold, *p* = 0.002) were downregulated compared to in control UL cells. After treatment with miR-197 inhibitor in UL cells, the relative expression levels of P-Rcα (14.324-fold, *p* = 0.008), MMP-1 (15.127-fold, *p* = 0.018), MMP-2 (20.512-fold, *p* = 0.004), MMP-9 (1.691-fold, *p* = 0.035), and TIMP-2 (5.522-fold, *p* < 0.001) were upregulated compared to in control UL cells.

After transfection of miR-200c mimic into UL cells, the relative expression levels of P-Rcα (0.024-fold, *p* = 0.009), P-Rcβ (0.003-fold, *p* < 0.001), E-Rcα (0.513-fold, *p* = 0.020), E-Rcβ (0.603-fold, *p* = 0.021), MMP-1 (0.265-fold, *p* = 0.002), and MMP-2 (0.800-fold, *p* = 0.037) were downregulated compared to in control UL cells. After treatment with miR-200c inhibitor in UL cells, the relative expression levels of E-Rcα (16.070-fold, *p* = 0.008), E-Rcβ (8.697-fold, *p* = 0.002), MMP-1 (43.130-fold, *p* = 0.010), MMP-2 (10.308-fold, *p* = 0.002), and MMP-9 (2.820-fold, *p* = 0.022) were upregulated compared to in control UL cells.

### 2.5. The Expression Levels of miRNAs and Candidate Target Genes Associated to Endometrial Cavity Distortion (ECD)

**Figure 6:**
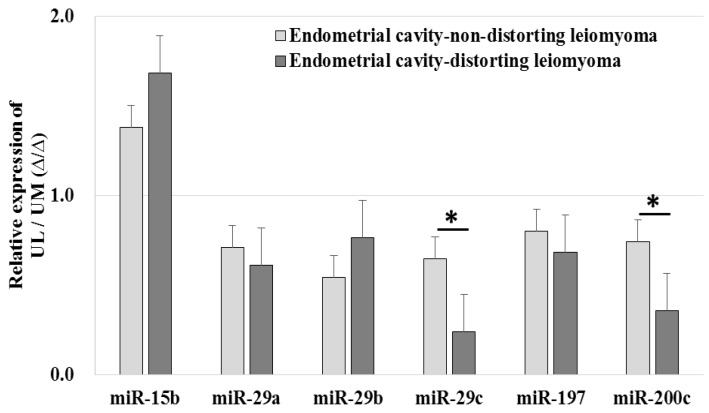
The comparison of relative miRNA expression in endometrial cavity-distorting and endometrial cavity-non-distorting leiomyoma cells compared to uterine myometrial cells (* *p* > 0.05).

The relative expression levels of miR-29c (0.240 ± 0.095-fold vs. 0.650 ± 0.277-fold, *p* < 0.001) and miR-200c (0.357 ± 0.156-fold vs. 0.745 ± 0.408-fold, *p* = 0.002) were downregulated in ECDL cells compared to in ECNDL cells (Figure 6).

**Figure 7:**
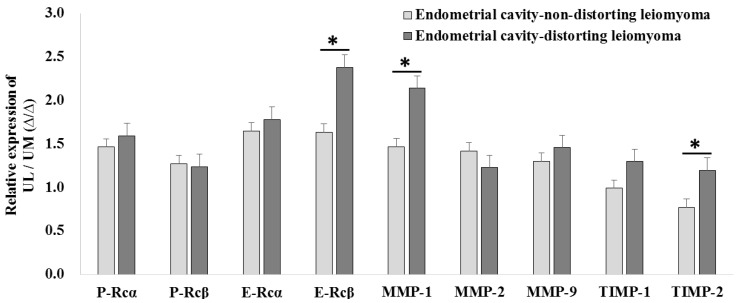
The comparison of relative candidate target gene expression in endometrial cavity-distorting and endometrial cavity-non-distorting leiomyoma cells compared to uterine myometrial cells (* *p* > 0.05).

The relative expression levels of E-Rcβ (2.380 ± 1.042-fold vs. 1.636 ± 0.496-fold, *p* = 0.028), MMP-1 (2.139 ± 0.710-fold vs. 1.465 ± 0.381-fold, *p* = 0.008), and TIMP-2 (1.197 ± 0.245-fold vs. 0.770 ± 0.459-fold, *p* = 0.004) were upregulated in ECDL cells compared to in ECNDL cells (Figure 7).

## 3. Discussion

ULs are the most common benign tumors of the uterus among women of reproductive age [20]. ULs might affect conception and pregnancy maintenance owing to the occurrence of ECD, and numerous treatment options have been employed, including surgery and in vitro fertilization [21,22,23,24,25,26]. The role of microRNA has been suggested in the development of leiomyoma as well as pregnancy-related complications [27,28,29,30]. The present study aimed to evaluate the profile of miRNA expression in UL cells with respect to their ability to cause the distortion of endometrial cavity.

This study demonstrated the upregulated expression level of miR-15b and downregulated expression levels of miR-29a, -29b, -29c, -197, and -200c in UL cells compared to in the matched normal UM cells. In addition to qPCR, which suggested an upregulation of miR-15b levels, in situ hybridization was performed to detect its localization in UL tissues. These results were consistent with those of previous studies [18,31,32,33]. Several investigators reported that the marked upregulation in miR-15b expression levels in UL cells might modulate numerous cellular biological processes such as cell proliferation, division, apoptosis, migration, invasion, metabolism, stress, angiogenesis, and drug resistance [34,35,36]. The miR-29 family is related to the accumulation and remodeling of the extracellular matrix (ECM) [18,37], which is believed to be crucial during the pathophysiology of ULs. miR-197 and -200 are known to regulate cell proliferation and suppress tumor development [32,38,39].

In candidate target gene analysis, we found that the gene expression of hormonal receptors and regulators of ECM was upregulated in UL cells compared to in UM cells. Consistent with our results, previous studies reported the upregulated expression of these genes in UL tissues compared to in the matched UM tissues [40,41,42]. In our study, immunofluorescence analysis and transfection experiments using miRNA analogues suggested that these miRNAs might not only regulate genes that modulate the composition of ECM such as MMPs and TIMPs, but might also play a role in the alteration of hormonal activities via modifying the gene expression of estrogen and progesterone receptors. Consistent with our results, a few previous reports suggested that these miRNAs affect the receptivity of estrogen and progesterone receptors [43,44,45,46,47]. Our results suggest that miRNAs might play a role in the development of ULs via the regulation of the hormonal micro-environment and composition of ECM.

**Figure 8:**
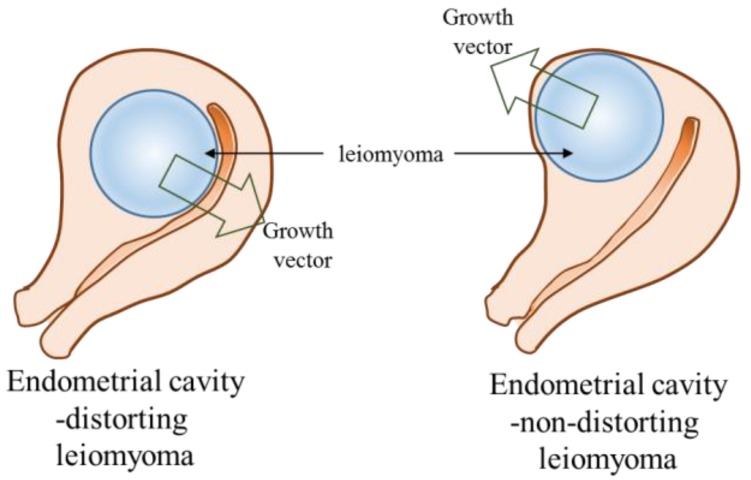
Diagram of endometrial cavity-distorting and endometrial cavity-non-distorting leiomyoma in intramural uterine leiomyoma (arrow: growth vector).

In our results, ECDL cells exhibited a dissimilar miRNA expression profile to ECNDL cells. Although both types of UL cells exhibited downregulated expression levels of miR-29b and -200c compared to those in matched UM cells, ECDL cells manifested a greater downregulation of the expression levels of these miRNAs compared to in ECNDL cells. Even though a significant variation in the expression levels of most of the target genes was not observed, the expression levels of E-Rcβ, MMP-1, and TIMP-2 were upregulated in ECDL cells compared to in ECNDL cells. These results suggest that miR-29b and -200c might regulate the expression of E-Rcβ, MMP-1, and TIMP-2 in UL cells and they might play a discriminatory role during ECD process in the uterus (Supplementary Figure S1). Previous studies reported that these genes were upregulated in UL cells compared to in matched UM cells [48,49,50]. A previous report demonstrated a significant variation in the expression levels of MMP-1 in UL cells compared to in UM cells depending on their size [50]. Corroborating these previous studies, our results suggest that ECDLs might exhibit varied composition profiles, miRNA regulation, and resultant growth pattern, with a contrary growing vector compared to ECNDLs (Figure 8). However, caution should be taken when interpreting our data since they could not fully explain the causality of miRs with regard to the growth direction of UL, although their association was suggested.

ULs might disrupt the anatomical structure of the normal uterus. Particularly, ECDLs might affect the endometrial receptivity, which in turn affects the embryo implantation and pregnancy maintenance. However, the etiology of ULs remains unknown. Furthermore, to the best of our knowledge, previous studies did not report on the profile of miRNAs in UL cells with respect to their ability to cause ECD. Present results implicate that the ability of UL cells to structurally deform the endometrial cavity might vary with respect to their miRNA expression profiles, even if they manifested a similar size and location in the uterine matrix. The regulation of a miRNA profile that induces UL cells to distort the uterine anatomical structure might be applied as a novel therapeutic strategy for ULs. However, further study is necessary to understand the mechanism by which UL cells acquire the ability to distort the normal uterine structure.

## 4. Materials and Methods

### 4.1. Sample Collection

In this study, women (*n* = 26) with single intramural UL were recruited after acquiring their consent. Pre-operative ultrasonography was performed to detect endometrial cavity-non-distorting leiomyoma (ECNDL, *n* = 15) and endometrial cavity-distorting leiomyoma (ECDL, *n* = 11) cases among these women. Sufficient amount of UL tissue along with adjacent normal UM tissue was collected from each patient by performing laparoscopic myomectomy and processed to obtain pathological confirmation. During the three months prior to surgery, gonadotropin-releasing hormone (GnRH) analogues and hormones such as estrogen and progestin were not used by the patients. This study was approved by the Institutional Review Board (KUGH17183-001 as of 7 September 2017).

### 4.2. Measurement of Hardness of Uterine Leiomyoma (UL) Tissues 

After UL tissue collection, hardness of each UL tissue was assessed by assigning non-parametric grade based on the area measurement of an indentation created by a Hegar dilator (No. 8) with a constant force applied for 30 s. The hardness grading was as follows: 1+ indentation diameter > 8 mm; 2+ indentation diameter ≤ 8 mm; 3+ no indentation.

### 4.3. RNA Isolation from Uterine Leiomyoma (UL) and Uterine Myometrium (UM) Tissues

Total RNA was isolated from each UL tissue and its corresponding (matched) UM tissue. Briefly, the tissues were washed with pre-warmed Hank’s balanced salt solution (HBSS: Invitrogen, Grand Island, NY, USA) and cut into 1 x 1 mm^2^ pieces using a surgical blade (No. 11: Feather safety razor, Osaka, Japan). Tissue pieces were collected into 5-mL microtubes and incubated with Trizol (Invitrogen) for 15 min at room temperature. During incubation, tissues were resuspended using constant pipetting and total RNA isolation was performed according to the manufacturer’s protocol.

### 4.4. qRT-PCR to Assess the Levels of miRNAs

cDNAs were synthesized from 0.5 µg of total RNA using miScript II RT Kit (Qiagen, Germantown, MD, USA) and were used as templates for qPCR reactions. specific primers (Table 3) and cDNAs were mixed with NCode™ Express SYBR^®^ GreenER™ miRNA qPCR premix (Invitrogen) and amplified under the following conditions: initial incubation for 2 min at 50 °C, followed by 2 min at 95 °C, and then, 40 cycles of 15 s at 95 °C, and 60 s at 60 °C. All the reactions were performed in triplicates and the Ct value was calculated based on the U6 expression, as follows.

(1) $$\mathrm{Relative}\mathrm{expression}(\Delta /\Delta )=\frac{\left(\mathrm{miR}\mathrm{expression}\mathrm{in}\mathrm{uterine}\mathrm{leiomyoma}\mathrm{cell})/(U6\mathrm{expression}\mathrm{in}\mathrm{uterine}\mathrm{leiomyoma}\mathrm{cell}\right)}{\left(\mathrm{miR}\mathrm{expression}\mathrm{in}\mathrm{uterine}\mathrm{myometrial}\mathrm{cell})/(U6\mathrm{expression}\mathrm{in}\mathrm{uterine}\mathrm{myometrial}\mathrm{cell}\right)}$$

### 4.5. qPCR to Assess the Levels of Candidate Target Genes

cDNAs were synthesized from 0.5 µg of total RNA using Accute RT-premix (Bioneer, Daejeon, Korea) and were mixed with QuantiTect SYBR green PCR premix (Qiagen) and specific primers. The amplification program included an initial step at 95 °C for 15 min, followed by 45 cycles of denaturation at 95 °C for 15 s, annealing at 58 °C for 20 s, and extension at 72 °C for 30 s. All the reactions were run in triplicates and the relative gene expression was normalized using the corresponding GAPDH expression as follows. The specific primers used for qPCR are shown in Table 4.

(2) $$\mathrm{Relative}\mathrm{expression}(\Delta /\Delta )=\frac{\left(\mathrm{Gene}\mathrm{expression}\mathrm{in}\mathrm{uterine}\mathrm{leiomyoma}\mathrm{cell})/(\mathrm{GAPDH}\mathrm{expression}\mathrm{in}\mathrm{uterine}\mathrm{leiomyoma}\mathrm{cell}\right)}{\left(\mathrm{Gene}\mathrm{expression}\mathrm{in}\mathrm{uterine}\mathrm{myometrial}\mathrm{cell})/(\mathrm{GAPDH}\mathrm{expression}\mathrm{in}\mathrm{uterine}\mathrm{myometrial}\mathrm{cell}\right)}$$

### 4.6. In Situ Hybridization of miRNAs

All the soluble reagents and products used in the in situ experiments were either pre-treated with diethylpyrocarbonate (DEPC) or diluted with DEPC-water (VWR, Radnor, PA, USA) to avoid contamination of DNA, DNase and RNase. miRCURY LNA™ control and specific probes were purchased from Exiqon (Germantown, MD, USA). To visualize the expression of specific miRNA in UL tissues, they were fixed in 10% formalin solution (Sigma-Aldrich, St. Louis, MO, USA) for 24 h and cut into 2 × 2 cm^2^. Fixed tissues were transferred into paraffin wax and allowed to solidify to form paraffin blocks. Solidified tissue blocks were cut to 5 µm thickness using a microtome (Leica, Biosystems, Wetzlar, Germany) and the sections were transferred onto microslides. These slides were dried at RT for 24 h. The prepared glass slides were deparaffinized at 60 °C and rehydrated prior to their use.

During the in situ hybridization process, the prepared slides were incubated in 20 μg/mL proteinase-K solution for 10 min at 37 °C and washed with phosphate-buffered saline (PBS). A hybridization mix solution containing in situ hybridization buffer in addition to either 40 nM miR-15b-FITC conjugated specific probes or 10 nM control probes was hybridized for 1 h at 58 °C. After hybridization, the prepared slides were serially washed with saline–sodium citrate (SSC) buffer (Invitrogen) and incubated with blocking solution (2% sheep serum) for 15 min at room temperature. The sample slides were washed and counter stained with 5 mg/mL of 4′,6-diamidino-2-phenylindole (DAPI, Invitrogen) for 20 min at room temperature and observed using an EVOS-FL fluorescence microscope.

The positive and negative control slides were washed and anti-Digoxin (DIG) reagent (Roche Diagnostics, Rotkreuz, Switzerland) was applied for 1 h at room temperature. After incubation, all the slides were washed three times using Phosphate buffered saline-Tween-20 (PBS-T, PBS containing 0.1% Tween-20) and incubated with alkaline phosphatase (AP) substrate (Vector Laboratories, Burlingame, CA, USA) for 2 h at 30 °C in a humidifying chamber. To stop the reaction, AP stop solution was applied for 5 min at RT. Finally, slides were counter stained with Nuclear Fast Red (Vector Laboratories) for 10 min at RT and observed using EVOS-FL fluorescence microscope (Thermo Fisher Scientific, Waltham, MA, USA).

### 4.7. Culture of UL Cells

UL tissue pieces were treated with 1 mg/mL of collagenase type I (Invitrogen) for 1 h at 37 °C in a water bath and repeatedly suspended by gentle pipetting. The digested tissues were filtered through a 70-µm cell strainer (SPL Life Sciences, Seoul, Korea) and centrifuged at 3000 rpm. Cell pellets were replated and the medium was replaced with fresh medium every other day. The medium consisted of 1:1 mix of Dulbecco’s modified Eagle medium and Ham’s F-12 medium (DMEM/F12) without phenol red, 10% fetal bovine serum (FBS), 1% insulin–transferrin–selenium (ITS), and 50 U/mL penicillin–streptomycin. All these reagents were purchased from Invitrogen.

### 4.8. Transfection of Mimics or Inhibitors of miRNAs into In Vitro Cultured UL Cells

Specific miRNA mimics and inhibitors were purchased from Genolution (Seoul, Korea) according to total miRNA sequence (available online: http://www.mirbase.org); 10 µM of each mimic or inhibitor was transfected into cultured UL cells using Lipofectamine RNAiMAX transfection reagent (Thermo Fisher Scientific) and the cells were harvested for RNA isolation after 48 h. UL cells transfected solely with transfection reagent without miRNA mimics or inhibitors were used as controls.

### 4.9. Statistical Analysis

All the experiments were independently repeated thrice using a distinct portion of each tissue sample. The variations were compared between UL and UM groups, and then between ECDL and ECNDL groups. The statistical significance was determined using Student’s *t-*test. The variations among the values of mean and standard deviation were compared using the Student’s t test. Non-parametric variables were analyzed using a chi-square test. Variations were considered statistically significant if the *p* value was < 0.05. All data were analyzed using the Statistical Package for the Social Sciences in Windows software (version 12.0, SPSS Inc., Chicago, IL, USA).

## 5. Conclusions

In conclusion, the expression profile of miRNAs in UL cells was different from that in the matched UM cells. Furthermore, the expression profile of miRNAs in UL cells varied with respect to their ability to cause ECD in the uterus. The miRNA profile might regulate the biomechanical properties of ULs to affect the structural homeostasis of the uterus. Therefore, targeting these specific miRNAs and their target genes may assist in the treatment of UL patients.

## Figures and Tables

**Table 1:** Comparison of clinical characteristics.

Characteristics	Endometrial Cavity-Non-Distorting Leiomyoma (*n* = 15)	Endometrial Cavity-Distorting Leiomyoma (*n* = 11)
Age (years)	32.5 ± 3.2	31.7 ± 4.3
Size of leiomyoma (maximal diameter, cm)	4.2 ± 1.3	4.5 ± 0.9
Chief complaints		
Dysmenorrhea	7/15 (46.7%)	8/11 (72.7%)
Menorrhagia	9/15 (60.0%)	9/11 (81.8%)
Infertility	15/15 (100%)	11/11 (100%)

*p* > 0.05 in all.

**Table 2:** Comparison of tissue hardness.

Tissue Hardness *	1+	2+	3+	Total
Endometrial cavity-non-distorting leiomyoma (n)	2	6	7	15
Endometrial cavity-distorting leiomyoma (n)	3	5	3	11

* 1+ indentation left by an indenter for over 10 min. 2+ indentation left by an indenter within 10 min. 3+ no indentation left by an indenter. Chi-square analysis, *p* > 0.05.

**Table 3:** Sequences of miRNAs.

miRNA	Sequence
U6	gtgctcgcttcggcagcacatatac
miR-15b	tagcagcacatcatggtttaca
miR-29a	tagcaccatctgaaatcggtt
miR-29b	tagcaccatttgaaatcagtgtt
miR-29c	tagcaccatttgaaatcggt
miR-197	ttcaccaccttctccacccagc
miR-200c	taatactgccgggtaatgatgga

**Table 4:** Sequences of target genes.

Gene	Forward Sequence	Reverse Sequence
*P-Rcα*	GAGCACTGGATGCTGTTGCT	GGCTTAGGGCTTGGCTTTC
*P-Rcβ*	TGGGATCTGAGATCTTCGGAG	GAAGGGTCGGACTTCTGCTG
*E-Rcα*	TACTGACCAACCTGGCAGACAG	TGGACCTGATCATGGAGGGT
*E-Rcβ*	AGTTGGCCGACAAGGAGTTG	CGCACTTGGTCGAACAGG
*MMP-1*	ACGGATACCCCAAGGACATCT	TCAGAAAGAGCATCGATATG
*MMP-2*	GGACACACTAAAGAAGATGCAGAAGT	CGCATGGTCTCGATGGTATTC
*MMP-9*	CCCGGAGTGAGTTGAACCA	GGATTTACATGGCACTGCC
*TIMP-1*	CTGCGGATACTTCCACAGGTC	GCAAGAGTCCATCCTGCAGTT
*TIMP-2*	ATAAGCAGGCCTCCAACGC	GAGCTGGACCAGTCGAAACC

*P-Rcα*: progesterone receptor α, *P-Rcβ*: progesterone receptor β, *E-Rcα*: estrogen receptor α, *E-Rcβ*: estrogen receptor β, MMP: matrix metalloproteinase, TIMP: tissue inhibitors of metalloproteinases.

## Supplementary Materials

Supplementary materials can be found at http://www.mdpi.com/1422-0067/19/9/2524/s1.

## References

[1] Al-Hendy A., Salama S. (2006). Gene Therapy and uterine leiomyoma: A review. Hum. Reprod. Update.

[2] Drayer S.M., Catherino W.H. (2015). Prevalence, morbidity, and current medical management of uterine leiomyomas. Int. J. Gynaecol. Obstet..

[3] Benson C.B., Chow J.S., Chang-Lee W., Hill J.A., Doubilet P.M. (2001). Outcome of pregnancies in women with uterine leiomyomas identified by sonography in the first trimester. J. Clin. Ultrasound.

[4] Hart R., Khalaf Y., Yeong C.T., Seed P., Taylor A., Braude P. (2001). A Prospective controlled study of the effect of intramural uterine fibroids on the outcome of assisted conception. Hum. Reprod..

[5] Casini M.L., Rossi F., Agostini R., Unfer V. (2006). Effects of the position of fibroids on fertility. Gynecol. Endocrinol..

[6] Pritts E.A., Parker W.H., Olive D.L. (2009). Fibroids and infertility: An updated systematic review of the evidence. Fertil. Steril..

[7] Bartel D.P. (2009). MicroRNAs: Target recognition and regulatory functions. Cell.

[8] Sokol N.S., Ambros V. (2005). Mesodermally expressed drosophila microRNA-1 is regulated by twist and is required in muscles during larval growth. Genes Dev..

[9] Poy M.N., Eliasson L., Krutzfeldt J., Kuwajima S., Ma X., MacDonald P.E., Pfeffer S., Tuschl T., Rajewsky N., Rorsman P. (2004). A pancreatic islet-specific microRNA regulates insulin secretion. Nature.

[10] Lai E.C. (2005). MiRNAs: Whys and wherefores of miRNA-mediated regulation. Curr. Biol..

[11] He L., Hannon G.J. (2004). MicroRNAs: Small RNAs with a big role in gene regulation. Nat. Rev. Genet..

[12] Boutet S., Vazquez F., Liu J., Béclin C., Fagard M., Gratias A., Morel J.B., Crété P., Chen X., Vaucheret H. (2003). Arabidopsis HEN1: A genetic link between endogenous miRNA controlling development and siRNA controlling transgene silencing and virus resistance. Curr. Biol..

[13] Kim Y.J., Ku S.Y., Kim Y.Y., Liu H.C., Chi S.W., Kim S.H., Choi Y.M., Kim J.G., Moon S.Y. (2013). MicroRNAs transfected into granulosa cells may regulate oocyte meiotic competence during in vitro maturation of mouse follicles. Hum. Reprod..

[14] Kim Y.J., Ku S.Y., Rosenwaks Z., Liu H.C., Chi S.W., Kang J.S., Lee W.J., Jung K.C., Kim S.H., Choi Y.M. (2010). MicroRNA expression profiles are altered by gonadotropins and vitamin c status during in vitro follicular growth. Reprod. Sci..

[15] Kim Y.J., Ku S.Y., Kim Y.Y., Suh C.S., Kim S.H., Choi Y.M. (2016). MicroRNA profile of granulosa cells after ovarian stimulation differs according to maturity of retrieved oocytes. Geburtshilfe Frauenheilkd..

[16] Kim Y.Y., Min H., Kim H., Choi Y.M., Liu H.C., Ku S.Y. (2017). Differential microRNA expression profile of human embryonic stem cell-derived cardiac lineage cells. Tissue Eng. Regen. Med..

[17] Chuang T.D., Khorram O. (2018). Expression profiling of lncRNAs, miRNAs, and mRNAs and their differential expression in leiomyoma using next-generation RNA sequencing. Reprod. Sci..

[18] Marsh E.E., Steinberg M.L., Parker J.B., Wu J., Chakravarti D., Bulun S.E. (2016). Decreased expression of microRNA-29 family in leiomyoma contributes to increased major fibrillar collagen production. Fertil. Steril..

[19] Pan Q., Luo X., Chegini N. (2010). MicroRNA 21: Response to hormonal therapies and regulatory function in leiomyoma, transformed leiomyoma and leiomyosarcoma cells. Mol. Hum. Reprod..

[20] Parker W.H. (2007). Etiology, symptomatology, and diagnosis of uterine myomas. Fertil. Steril..

[21] Brady P.C., Stanic A.K., Styer A.K. (2013). Uterine fibroids and subfertility: An update on the role of myomectomy. Curr. Opin. Obstet. Gynecol..

[22] Lagana A.S., Vergara D., Favilli A., La Rosa V.L., Tinelli A., Gerli S., Noventa M., Vitagliano A., Triolo O., Rapisarda A.M.C. (2017). Epigenetic and genetic landscape of uterine leiomyomas: A current view over a common gynecological disease. Arch. Gynecol. Obstet..

[23] Vitale S.G., Sapia F., Rapisarda A.M.C., Valenti G., Santangelo F., Rossetti D., Chiofalo B., Sarpietro G., La Rosa V.L., Triolo O. (2017). Hysteroscopic morcellation of submucous myomas: A systematic review. Biomed. Res. Int..

[24] Choi Y.S., Ku S.Y., Jee B.C., Suh C.S., Choi Y.M., Kim J.G., Moon S.Y., Kim S.H. (2006). Comparison of follicular fluid IGF-i, IGF-ii, IGFBP-3, IGFBP-4 and PAPP-a concentrations and their ratios between gnrh agonist and GNRH antagonist protocols for controlled ovarian stimulation in ivf-embryo transfer patients. Hum. Reprod..

[25] Kim Y.J., Ku S.Y., Jee B.C., Suh C.S., Kim S.H., Choi Y.M., Kim J.G., Moon S.Y. (2010). A comparative study on the outcomes of in vitro fertilization between women with polycystic ovary syndrome and those with sonographic polycystic ovary-only in gnrh antagonist cycles. Arch. Gynecol. Obstet..

[26] Ku S.Y., Suh C.S., Kim S.H., Choi Y.M., Kim J.G., Moon S.Y. (2003). A pilot study of the use of low dose human menopausal gonadotropin in ovulation induction. Eur. J. Obstet. Gynecol. Reprod. Biol..

[27] Karmon A.E., Cardozo E.R., Rueda B.R., Styer A.K. (2014). MicroRNAs in the development and pathobiology of uterine leiomyomata: Does evidence support future strategies for clinical intervention?. Hum. Reprod. Update.

[28] Georgieva B., Milev I., Minkov I., Dimitrova I., Bradford A.P., Baev V. (2012). Characterization of the uterine leiomyoma microRNAome by deep sequencing. Genomics.

[29] Chiofalo B., Lagana A.S., Vaiarelli A., La Rosa V.L., Rossetti D., Palmara V., Valenti G., Rapisarda A.M.C., Granese R., Sapia F. (2017). Do miRNAs play a role in fetal growth restriction? a fresh look to a busy corner. Biomed. Res. Int..

[30] Lycoudi A., Mavreli D., Mavrou A., Papantoniou N., Kolialexi A. (2015). MiRNAs in pregnancy-related complications. Expert. Rev. Mol. Diagn..

[31] Guan Y., Guo L., Zukerberg L., Rueda B.R., Styer A.K. (2016). MicroRNA-15b regulates reversion-inducing cysteine-rich protein with kazal motifs (reck) expression in human uterine leiomyoma. Reprod. Biol. Endocrinol..

[32] Wu X., Ling J., Fu Z., Ji C., Wu J., Xu Q. (2015). Effects of miRNA-197 overexpression on proliferation, apoptosis and migration in levonorgestrel treated uterine leiomyoma cells. Biomed. Pharmacother..

[33] Chuang T.D., Khorram O. (2014). Mir-200c regulates Il8 expression by targeting IKBKB: A potential mediator of inflammation in leiomyoma pathogenesis. PLoS ONE.

[34] Xia L., Zhang D., Du R., Pan Y., Zhao L., Sun S., Hong L., Liu J., Fan D. (2008). MiR-15b and miR-16 modulate multidrug resistance by targeting *bcl2* in human gastric cancer cells. Int. J. Cancer.

[35] Oh J., Takahashi R., Kondo S., Mizoguchi A., Adachi E., Sasahara R.M., Nishimura S., Imamura Y., Kitayama H., Alexander D.B. (2001). The membrane-anchored mmp inhibitor reck is a key regulator of extracellular matrix integrity and angiogenesis. Cell.

[36] Wang X., Tang S., Le S.Y., Lu R., Rader J.S., Meyers C., Zheng Z.M. (2008). Aberrant expression of oncogenic and tumor-suppressive microRNAs in cervical cancer is required for cancer cell growth. PLoS ONE.

[37] Qiang W., Liu Z., Serna V.A., Druschitz S.A., Liu Y., Espona-Fiedler M., Wei J.J., Kurita T. (2014). Down-regulation of mir-29b is essential for pathogenesis of uterine leiomyoma. Endocrinology.

[38] Li X., Roslan S., Johnstone C.N., Wright J.A., Bracken C.P., Anderson M., Bert A.G., Selth L.A., Anderson R.L., Goodall G.J. (2014). MiR-200 can repress breast cancer metastasis through zeb1-independent but moesin-dependent pathways. Oncogene.

[39] Kim J.S., Kurie J.M., Ahn Y.H. (2015). Bmp4 Depletion by MiR-200 inhibits tumorigenesis and metastasis of lung adenocarcinoma cells. Mol. Cancer.

[40] Bogusiewicz M., Stryjecka-Zimmer M., Postawski K., Jakimiuk A.J., Rechberger T. (2007). Activity of matrix metalloproteinase-2 and -9 and contents of their tissue inhibitors in uterine leiomyoma and corresponding myometrium. Gynecol. Endocrinol..

[41] Ishikawa H., Ishi K., Serna V.A., Kakazu R., Bulun S.E., Kurita T. (2010). Progesterone is essential for maintenance and growth of uterine leiomyoma. Endocrinology.

[42] Ishikawa H., Reierstad S., Demura M., Rademaker A.W., Kasai T., Inoue M., Usui H., Shozu M., Bulun S.E. (2009). High aromatase expression in uterine leiomyoma tissues of African-American women. J. Clin. Endocrinol. Metab..

[43] Cittelly D.M., Finlay-Schultz J., Howe E.N., Spoelstra N.S., Axlund S.D., Hendricks P., Jacobsen B.M., Sartorius C.A., Richer J.K. (2013). Progestin suppression of mir-29 potentiates dedifferentiation of breast cancer cells via *klf4*. Oncogene.

[44] Vares G., Sai S., Wang B., Fujimori A., Nenoi M., Nakajima T. (2015). Progesterone generates cancer stem cells through membrane progesterone receptor-triggered signaling in basal-like human mammary cells. Cancer Lett..

[45] Muluhngwi P., Richardson K., Napier J., Rouchka E.C., Mott J.L., Klinge C.M. (2017). Regulation of miR-29b-1/a transcription and identification of target mRNAs in cho-k1 cells. Mol. Cell. Endocrinol..

[46] Kim Y.Y., Kim Y.J., Cho K.M., Kim S.H., Park K.E., Kang B.C., Jung K.C., Kim M.S., Ku S.Y. (2016). The expression profile of angiotensin system on thawed murine ovaries. Tissue Eng. Regen. Med..

[47] Kim Y.J., Kim Y.Y., Kim D.W., Joo J.K., Kim H., Ku S.Y. (2017). Profile of microRNA expression in endometrial cell during in vitro culture according to progesterone concentration. Tissue Eng. Regen. Med..

[48] Tian R., Wang Z., Shi Z., Li D., Wang Y., Zhu Y., Lin W., Gui Y., Zheng X.L. (2013). Differential expression of g-protein-coupled estrogen receptor-30 in human myometrial and uterine leiomyoma smooth muscle. Fertil. Steril..

[49] Morikawa A., Ohara N., Xu Q., Nakabayashi K., DeManno D.A., Chwalisz K., Yoshida S., Maruo T. (2008). Selective progesterone receptor modulator asoprisnil down-regulates collagen synthesis in cultured human uterine leiomyoma cells through up-regulating extracellular matrix metalloproteinase inducer. Hum. Reprod..

[50] Wolanska M., Sobolewski K., Bankowski E., Jaworski S. (2004). Matrix metalloproteinases of human leiomyoma in various stages of tumor growth. Gynecol. Obstet. Investig..

